# Vitamin D: A Critical Regulator of Intestinal Physiology

**DOI:** 10.1002/jbm4.10554

**Published:** 2021-10-06

**Authors:** Sylvia Christakos

**Affiliations:** ^1^ Department of Microbiology, Biochemistry and Molecular Genetics Rutgers, the State University of New Jersey, New Jersey Medical School Newark NJ USA

**Keywords:** ANIMAL MODELS, CALCIUM, INTESTINE, RICKETS, VITAMIN D

## Abstract

Calcium is required for the functioning of numerous biological processes and is essential for skeletal health. The major source of new calcium is from the diet. The central role of vitamin D in the maintenance of calcium homeostasis is to increase the absorption of ingested calcium from the intestine. The critical importance of vitamin D in this process is noted in the causal link between vitamin D deficiency and rickets, as well as in studies using genetically modified mice including mice deficient in the vitamin D receptor (*Vdr* null mice) or in the cytochrome P‐450 enzyme, 25‐hydroxyvitamin D_3_‐1α‐ hydroxylase (CYP27B1) that converts 25‐hydroxyvitamin D_3_ to the hormonally active form of vitamin D, 1,25‐dihydroxyvitamin D_3_ [1,25(OH)_2_D_3_] (*Cyp27b1* null mice). When these mice are fed diets with high calcium and lactose, rickets is prevented. The studies using mouse models provide supporting evidence indicating that the major physiological function of 1,25(OH)_2_D_3_/VDR is intestinal calcium absorption. This review summarizes what is known about mechanisms involved in vitamin D‐regulated intestinal calcium absorption. Recent studies suggest that vitamin D does not affect a single entity, but that a complex network of calcium‐regulating components is involved in the process of 1,25(OH)_2_D_3_‐mediated active intestinal calcium absorption. In addition, numerous 1,25(OH)_2_D_3_ actions in the intestine have been described independent of calcium absorption. Although the translatability to humans requires further definition, an overview is presented that provides compelling evidence from the laboratory of 1,25(OH)_2_D_3_ intestinal effects, which include the regulation of adhesion molecules to enhance barrier function, the regulation of intestinal stem cell function, cellular homeostasis of other divalent cations, the regulation of drug metabolizing enzymes, and anti‐inflammatory effects. © 2021 The Author. *JBMR Plus* published by Wiley Periodicals LLC on behalf of American Society for Bone and Mineral Research.

## Introduction

Vitamin D is an important nutrient whose metabolite, 1,25‐dihydroxyvitamin D_3_ [1,25(OH)_2_D_3_], regulates the integrity and function of the intestine. In addition to the regulation of intestinal calcium absorption, there is convincing evidence of other beneficial intestinal effects of 1,25(OH)_2_D_3_, including the regulation of intestinal stem cells and maintenance of barrier function, as well as anti‐inflammatory effects. Novel intestinal targets of 1,25(OH)_2_D_3_ have been identified that may provide new insight into potentially critical actions of vitamin D in the regulation of intestinal physiology.

## Vitamin D, the intestine, and calcium homeostasis

Calcium has numerous roles in biological systems, including bone formation, hormone secretion, and enzyme activation.^(^ ^1^ ^)^ To serve these multiple functions calcium, ingested in the diet, needs to be absorbed by the intestine. The major function of 1,25(OH)_2_D_3_, the hormonally active form of vitamin D, is to increase calcium absorption from the intestine.^(^ ^2^, ^3^ ^)^ 1,25(OH)_2_D_3_ is produced by two sequential hydroxylations of vitamin D mainly by CYP2R1 in the liver to produce 25‐hydroxyvitamin D3 [25(OH)D_3_]. 25(OH)D_3_ is then converted to 1,25(OH)_2_D_3_ in the kidney by CYP27B1. 1,25(OH)_2_D_3_ acts through the vitamin D receptor (VDR) to mediate the transcription of target genes through a multistep process (see Pike and Christakos^(^ ^4^ ^)^ for a review). Studies using genetically modified mice have played an increasingly important role in the basic mechanisms involved in the control of calcium homeostasis.^(^ ^5^ ^)^ Studies in *Vdr* null mice represent an animal model of vitamin D‐dependent rickets type 2A (VDDR2A, OMIM 277440). They develop hypocalcemia, hypophosphatemia, and rickets, which is accompanied by a decrease in the expression of the epithelial calcium channel TRPV6 and the calcium‐binding protein, calbindin‐D_9k_ in the intestine.^(^ ^6^, ^7^, ^8^ ^)^ When *Vdr* null mice and *Cyp27b1* null mice [animal models of vitamin D hydroxylase‐deficient rickets type 1A (VDDR1A, OMIM 264700), which have advanced rickets and undetectable 1,25(OH)_2_D_3_ levels] are fed a rescue diet with high calcium and lactose, rickets is prevented.^(^ ^9^, ^10^, ^11^, ^12^ ^)^ In addition, intestine epithelium‐specific transgenic expression of VDR in *Vdr* null mice results in the rescue of VDR‐dependent rickets.^(^ ^13^, ^14^ ^)^ Thus, although multiple pleiotropic effects of 1,25(OH)_2_D_3_ have been suggested, collectively these findings indicate for the first time—using KO and transgenic mouse models—that enhancement of intestinal calcium absorption is the major physiological action of 1,25(OH)_2_D_3_/VDR.

**Fig 1:**
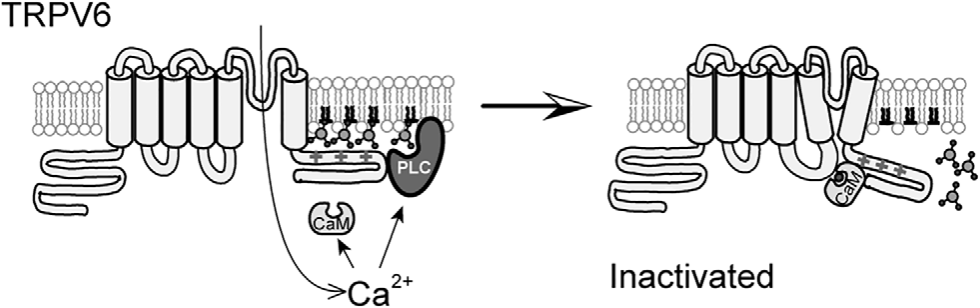
Model of Ca^2+^‐induced inactivation of TRPV6. Calcium‐calmodulin (CaM) inhibits TRPV6 activity via direct binding to the distal C‐terminal region. Ca^2+^ influx activates phospholipase C (PLC)‐mediated hydrolysis of PI(4,5)P_2_, which also contributes to Ca^2+^‐induced inactivation of TRPV6. Image courtesy of Tibor Rohacs, Rutgers New Jersey Medical School, Newark, NJ.

When there is an increased need for calcium (under low calcium conditions, during growth, pregnancy, or lactation), 1,25(OH)_2_D_3_ acts on the intestine to increase active calcium absorption. With regard to mechanisms involved, the traditional model suggests that the effect of vitamin D on calcium absorption is caused by the influx of calcium through the apical membrane calcium channel, TRPV6, the diffusion of calcium through the cytosol, which has been suggested to involve binding of calcium to the intracellular calcium‐binding protein calbindin‐D_9k_ (encoded by *S100g*), and active extrusion of calcium at the basolateral membrane by the plasma membrane ATPase (PMCA1b).^(^ ^15^ ^)^ However, gene‐deletion studies in mice, which showed that 1,25(OH)_2_D_3_‐mediated intestinal calcium absorption occurs in the absence of *Trpv6* and *S100g*, have challenged this traditional view.^(^ ^16^, ^17^, ^18^ ^)^ Although serum calcium is normal in *Trpv6* null mice (as well as *S100g* null mice), it should be noted that intestine‐specific transgenic expression of TRPV6 can increase intestinal calcium absorption and bone density in *Vdr* null mice, indicating that TRPV6 does have a direct role in the calcium absorptive process.^(^ ^19^ ^)^ The studies in the null mutant mice suggest that in the absence of TRPV6 and calbindin‐D_9k_ there is compensation by other, yet to be identified proteins. In addition, recent studies suggest that vitamin D does not affect a single entity, but that a complex network of calcium‐regulating components (e.g., calmodulin for fine‐tuning calcium channel activity and calcium binding to intracellular organelles, as well as other calcium‐binding proteins) is involved in the process of 1,25(OH)_2_D_3‐_mediated active intestinal calcium absorption.^(^ ^20^, ^21^ ^)^ Calcium‐binding components within the cell may contribute to sequestration of calcium, protecting against calcium‐mediated cytotoxicity. TRPV6 depends on phosphatidylinositol 4,5 bisphosphate [PI(4,5)P_2_] for activity.^(^ ^22^ ^)^ At high calcium concentrations, TRPV6 undergoes calcium‐induced inactivation. It has been suggested that calcium‐induced inactivation of TRPV6 may also be involved in protecting against the accumulation of toxic levels of calcium in the cell.^(^ ^22^ ^)^ Both calmodulin, which binds directly to the C terminal region of TRPV6 (see Fig. 1) and depletion of PI(4,5)P_2_ have been reported to contribute to Ca^2+^‐induced inactivation of TRPV6.^(^ ^22^ ^)^

Other proteins have been suggested to be involved in the process of intestinal calcium absorption. Recently, the L‐type calcium channel Ca_v_1.3 was proposed to mediate active calcium transport in the intestine. It was suggested that the actions of Ca_v_1.3 and TRPV6 are complementary.^(23^ ^)^ However, Ca_v_1.3 is not regulated by 1,25(OH)_2_D_3_.^(^ ^24^ ^)^ In addition, in mice fed a normal or low calcium diet, mRNA levels for Ca_v_1.3 are not related to changes in calcium absorption.^(^ ^25^ ^)^ It has been suggested that Ca_v_1.3 may mediate transcellular calcium absorption in the jejunum prior to weaning (which is prior to the induction of VDR at weaning).^(^ ^23^ ^)^ Therefore, a role for Ca_v_1.3 as an apical membrane calcium transporter that can contribute to vitamin D‐mediated calcium absorption is not supported by the in vivo data.^(^ ^23^, ^24^, ^25^ ^)^ In addition, TRPM7, a channel kinase, has recently been reported to be a central gatekeeper of intestinal absorption of magnesium, zinc, and calcium.^(^ ^26^ ^)^ It was proposed that TRPM7, and not TRPV6, is the key factor in intestinal calcium absorption.^(^ ^26^ ^)^ However, studies in mice with intestine‐specific knockout of *Trpm7* showing decreased serum levels of calcium were assessed at postnatal day 5, prior to the induction of intestinal VDR (mice died by postnatal day 10).^(^ ^26^ ^)^ In our recent transcriptomic analysis of 1,25(OH)_2_D_3_, genomic action in the intestine, *Trpm7* was not found to be regulated by 1,25(OH)_2_D_3._^(^ ^24^ ^)^ Thus, although TRPM7 may indeed be important for mineral absorption in early postnatal life, there is no evidence at this time of a role for TRPM7 in vitamin D‐mediated intestinal calcium absorption.

In addition to active transcellular calcium absorption, calcium is also absorbed by a paracellular pathway, a passive diffusional process that occurs through tight junctions and structures within intercellular spaces and predominates in the distal intestine when dietary calcium is high.^(^ ^15^ ^)^ Although intercellular adhesion molecules, tight junction channels, and proteins important for cell‐to‐cell contact were reported to be regulated in the intestine by vitamin D,^(^ ^27^, ^28^ ^)^ the physiological significance of their regulation by vitamin D with regard to calcium absorption has not been defined, and the role of vitamin D in the regulation of passive calcium transport remains a matter of debate. Epithelial tight junction proteins also regulate intestinal barrier function.^(^ ^29^ ^)^ A number of studies related to regulation by 1,25(OH)_2_D_3_/VDR of proteins involved in cell–cell adhesion have suggested an important role of VDR signaling to inhibit colitis by protecting against mucosal barrier dysfunction.^(^ ^30^, ^31^, ^32^, ^33^ ^)^ Thus, it is possible that the primary role of 1,25(OH)_2_D_3_‐mediated regulation of intercellular adhesion molecules is not to mediate passive calcium absorption, but rather to enhance barrier function.

## Vitamin D and intestinal stem cells

The action of 1,25(OH)_2_D_3_/VDR as a regulator of intestinal calcium absorption is well established. However, the effects of 1,25(OH)_2_D_3_ on intestinal stem cells are only beginning to be defined. Although previous studies have suggested that 1,25(OH)_2_D_3_‐mediated responses in the intestine occur at the villus or tip region rather than in the crypt region, we recently reported that the expression of *Vdr* and VDR protein in mouse villus and crypt are equivalent.^(^ ^24^ ^)^ Our findings are consistent with early autoradiographic studies showing that tritium‐labeled 1,25(OH)_2_D_3_ is concentrated in the intestine in absorptive as well as in crypt epithelial cells.^(^ ^34^ ^)^ In addition, using both isolated mouse villus and crypt, as well as human enteroids, we found that 1,25(OH)_2_D_3_ was able to regulate classic target genes in both villus and crypt.^(^ ^24^ ^)^ An important role of vitamin D signaling in the functioning of Lgr5^+^ intestinal stem cells, which contribute to intestinal homeostasis, was previously noted by L. Augenlicht's lab (Peregrina et al.^(^ ^35^ ^)^). Recent studies using human colon organoids noted coexpression of *LGR5* and *VDR* in crypt base‐columnar stem cells.^(^ ^36^ ^)^ In addition, stemness‐related genes including *MSI1*, which is important for crypt regeneration, were noted to be regulated by 1,25(OH)_2_D_3_ in normal organoids, but not in tumor organoids.^(^ ^36^ ^)^ 1,25(OH)_2_D_3_ was also reported to upregulate *LRIG1*, a master regulator of epithelial stem cells and a tumor suppressor, and to downregulate genes involved in colon carcinoma cell proliferation (*RARRES1* and *GRK5*).^(^ ^36^ ^)^ These findings further indicate an important regulatory role of vitamin D in the function of intestinal stem cells, which may contribute to intestinal homeostasis, and the suggested roles of vitamin D in protection against colorectal cancer^(^ ^37^ ^)^ and intestinal regeneration after injury.^(^ ^38^ ^)^

## A novel intestinal vitamin D target: the manganese efflux transporter, SLC30A10

**Fig 2:**
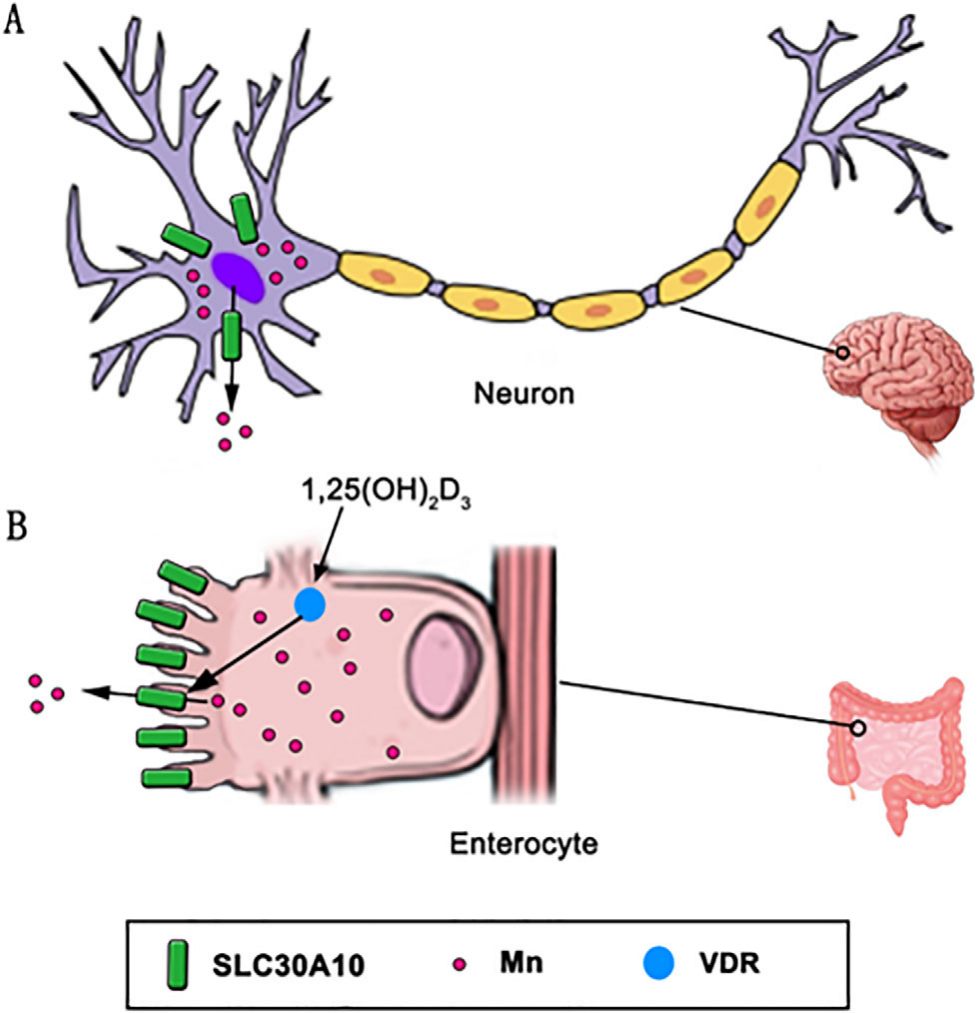
Regulation of SLC30A10 by 1,25‐dihydroxyvitamin D_3_ [1,25(OH)_2_D_3_] in the intestine. SLC30a10 localizes in neurons (A), in the apical domain of intestinal enterocytes (B), and hepatocytes (not shown); it transports Mn to the cell exterior, resulting in reduction of cellular Mn levels. A critical role for the intestine in Mn excretion has recently been reported.^(^ ^40^ ^)^ SLC30A10 in brain may also decrease Mn concentrations in brain during elevated Mn exposure.^(^ ^39^ ^)^ Vitamin D/1,25(OH)_2_D_3_ treatment may be a novel therapeutic strategy to increase Mn excretion from the intestine to prevent toxicity.^(^ ^24^ ^)^

In our recent studies related to 1,25(OH)_2_D_3_ genomic action in mouse intestine and human enteroids in addition to *Trpv6*, *S100g* and *Cyp24a1*, we found a manganese (Mn) efflux transporter, *Slc30a10*, was also one of the top genes induced by 1,25(OH)_2_D_3_ (Fig. 2).^(^ ^24^ ^)^ Mn is an essential element with a role in many physiological processes (including bone formation and brain function).^(^ ^39^ ^)^ At elevated levels caused by occupational or environmental exposure or diseases of the digestive system (e.g., cirrhosis), Mn accumulates in the brain and induces incurable neurotoxicity that manifests as Parkinsonism.^(^ ^39^ ^)^ SLC30A10 localizes to the apical domain of liver hepatocytes and intestinal enterocytes and transports Mn from the cytosol to the cell exterior, resulting in the reduction of cellular Mn levels and protection against toxicity.^(^ ^39^ ^)^ It is of interest that tissue Mn levels are modestly elevated in liver‐specific *Slc30a10* KO mice.^(^ ^40^ ^)^ However, when *Slc30a10* is deleted in both the liver and intestine, blood and brain Mn levels are markedly elevated, indicating a critical role for the intestine in Mn excretion.^(^ ^40^ ^)^ In *Slc30a10* KO mice, there is a marked decrease in the expression of *Trpv6* and *S100g* in the duodenum, suggesting that TRPV6, calbindin‐D_9k_, and SLC30A10 may work together in the intestine in Mn efflux transport.^(^ ^24^ ^)^ In addition, in recent in vitro studies it was noted that SLC30A10 is a secondary active transporter that uses calcium as the counter ion (i.e., transporting one calcium ion into cells, while exporting one Mn ion out of cells).^(^ ^41^ ^)^ Collectively, these findings suggest an interrelationship between the vitamin D endocrine system and intestinal SLC30A10 expression that directly influences Mn and calcium homeostasis. Currently, there are no available treatments for Mn‐induced neurological disease. It is possible that vitamin D/1,25(OH)_2_D_3_ treatment will be a novel therapeutic strategy to increase Mn excretion to prevent or manage Mn toxicity.^(^ ^24^, ^42^ ^)^

In addition to SLC30A10, our findings and that of others have noted that 1,25(OH)_2_D_3_ affects the expression of other intestinal transporters. We reported that SLC34A2, the intestinal cotransporter NaPi‐IIb is induced by 1,25(OH)_2_D_3_ in human enteroids derived from proximal intestine.^(^ ^24^ ^)^ 1,25(OH)_2_D_3_ is known to enhance intestinal phosphate absorption, as well as intestinal calcium absorption.^(^ ^21^ ^)^ However, the mechanisms involved in 1,25(OH)_2_D_3_‐mediated intestinal phosphate absorption have been a matter of debate. An age‐dependent regulation of NaPi‐IIb by 1,25(OH)_2_D_3_ in rats has been reported.^(^ ^43^ ^)^ In addition, intestinal stimulation of phosphate transport after 1,25(OH)_2_D_3_ administration to WT, but not to *Slc34a2*‐deficient mice has been observed.^(^ ^44^ ^)^ However, administration of a low phosphate diet to *Vdr* null mice resulted in a significant induction of NaPi‐IIb in the intestine, suggesting that there is adaptation to dietary phosphate content independent of vitamin D.^(^ ^45^ ^)^ In addition, studies in rats did not confirm the sodium dependence of 1,25(OH)_2_D_3_‐mediated intestinal phosphate transport.^(^ ^46^ ^)^ Further studies are needed to determine the physiological significance of the regulation of intestinal NaPi‐IIb by 1,25(OH)_2_D_3._ Previous studies also noted the induction by 1,25(OH)_2_D_3_ of several zinc (Zn) transporters (including those encoded by *Slc30a1*, *Slc30a5*) in the intestine.^(^ ^20^ ^)^ Similar to Mn, levels of Zn need to be maintained in a narrow range to prevent toxic effects. Zn is required for the activity of numerous enzymes and plays an important role in development, bone formation, and gene transcription, as well as in the immune system.^(^ ^47^ ^)^ Although early studies in chicks did not indicate a regulation of intestinal Zn absorption by vitamin D or 1,25(OH)_2_D_3,_^(^ ^48^ ^)^ because of the importance of both calcium and zinc in bone formation, further studies are needed to examine the interrelationship between the vitamin D endocrine system and the expression of the zinc transporters that may influence both Zn and calcium homeostasis.

## Xenobiotic metabolism

Mucosal epithelial cells express a number of enzymes involved in both endobiotic and xenobiotic metabolism. Previous studies have suggested that the liver was the major organ for xenobiotic metabolism.^(^ ^49^ ^)^ However, it has been reported that the intestine may contribute equally or more significantly than the liver.^(^ ^50^ ^)^ Drug metabolizing enzymes (DMEs) are involved in enzymatic oxidation (phase I enzymes) and conjugation (phase II enzymes). The cytochrome P450 enzymes are examples of phase I DMEs. Transcriptomic analysis using mucosa from the duodenum of vitamin D‐deficient rats injected with 1,25(OH)_2_D_3_ showed that 1,25(OH)_2_D_3_ stimulates the expression of a number of biotransformation enzyme genes including transcripts for CYP1A1 as well as for CYP3A1/CYP3A3 (the human homolog is CYP3A4).^(^ ^51^ ^)^ Enzymes of the CYP‐1, 2, and 3 families catalyze the majority of endobiotic and xenobiotic substrates.^(^ ^52^ ^)^ In our study using human enteroids (in collaboration with Z. Criss and N. Shroyer at Baylor College of Medicine, Houston, TX) transcripts for CYP3A4 (which acts on the greatest number of xenobiotics and is also involved in the vitamin D catabolic pathway) and CYP2C19 (an enzyme involved in a major pathway for oxidative modification of drugs) were noted as 1,25(OH)_2_D_3_ target genes.^(^ ^24^, ^50^, ^53^ ^)^ In addition to phase I enzymes, phase II enzymes (glucuronosyltransferases [UGTs] that catalyze glucuronidation reactions) were also found to be regulated by 1,25(OH)_2_D_3_ in the intestine.^(^ ^24^, ^51^ ^)^ It has been suggested that UGTs, by resulting in increased water solubility of their substrates through glucuronidation, facilitate absorption and targeting to the biliary tract for elimination.^(^ ^52^ ^)^ Thus, by regulating phase I and phase II biotransformation enzymes, 1,25(OH)_2_D_3_ may have an important role in protection from harmful environmental factors. In addition, regulation of these enzymes may provide another pathway for homeostatic control of the multiple actions of vitamin D.

## Immune response

1,25(OH)_2_D_3_ suppression of the production of IFNγ and IL‐17 by T cells and induction of regulatory T cells is known to protect against intestinal inflammation (see reviews by Cantorna, Rogers, et al.,^54^ Cantorna, Snyder, et al.,^55^ Wei and Christakos,^56^ and Bishop et al.^57^). In addition, the intestine contains specialized immune cells that maintain intestinal immune homeostasis. Intraepithelial lymphocytes (IELs) exist in contact with the intestinal epithelial cell layer and maintain immune homeostasis at the intestinal mucosal surface.^(^ ^58^ ^)^ T cells in the IELs of the small intestine (SI) express a homodimer of CD8α (CD8αα) IEC.^(^ ^55^, ^58^ ^)^ CD8αα+/CD4+ double‐positive T cells in the IEL are regulatory cells that produce IL‐10.^(^ ^59^ ^)^ In VDR KO mice, there are fewer of these CD4+CD8αα+T cells in the IEL compared with WT mice.^(^ ^60^, ^61^ ^)^ In addition, vitamin D deficiency results in fewer microbiota‐specific FoxP3+/RORγt+ regulatory T cells in the colon of mice.^(^ ^62^ ^)^ Fewer regulatory T cells in the SI IEL and the colon during vitamin D or VDR deficiency results in an imbalance in intestinal flora and an inflammatory response to normally nonpathogenic bacteria.^(^ ^60^ ^)^ Innate lymphoid cells (ILCs), found at the barrier surface of the intestine, also play an important role in intestinal homeostasis.^(^ ^58^ ^)^ ILC3 cells produce IL‐22, a protective cytokine that induces antimicrobial peptides, controls microbiota, and protects intestinal epithelia after injury or bacterial infection.^(^ ^58^ ^)^ Recent studies indicated the requirement of vitamin D for early IL‐22 production from ILC3 cells.^(^ ^63^ ^)^ In addition, 1,25(OH)_2_D_3_ treatment increased IL‐22 secreting ILC3 cells and improved resistance to *Citrobacter rodentium* infection of the gastrointestinal (GI) tract.^(^ ^63^ ^)^ It has been suggested that the regulation of microbiota in the GI tract by vitamin D may be an indirect effect based, in part, on the regulation by vitamin D of these specialized immune cells^(^ ^63^ ^)^ (see the review from the M. Cantorna lab.^(^ ^55)^)

The importance of vitamin D in intestinal immune homeostasis was further shown in mice with transgenic expression of VDR only in IECs, which resulted in the rescue of *Vdr* null mice from TNBS (2,4,6 trinitrobenzene sulfonic acid)‐induced colitis, as well as protection against colitis in WT mice.^(^ ^33^ ^)^ The mechanism involved in part inhibition of NF‐κB activation.^(^ ^33^ ^)^ In addition, in the IL‐10 KO model of colitis, intestinal epithelial VDR overexpression markedly reduced intestinal inflammation, as well as colonic crypt hyperplasia and lamina propria lymphocyte infiltration.^(^ ^64^ ^)^ The contribution of intestinal epithelial cells to intestinal inflammation is also noted in IEC‐specific *Rab11a* KO mice. Rab11a, a GTPase, is involved in membrane recycling and has been reported to control intestinal microbial tolerance.^(^ ^65^ ^)^ Genetic ablation of *Rab11a* (a gene adjacent to a Crohn disease locus) in mouse intestinal cells results in cell‐intrinsic cytokine production and an inflammatory bowel phenotype.^(^ ^66^ ^)^ 1,25(OH)_2_D_3_ treatment in addition to feeding *Rab11a* KO mice with high dietary vitamin D supplementation (20,000 IU/kg diet) decreased inflammatory cytokines and reduced crypt cell proliferation.^(^ ^67^ ^)^ The effects of 1,25(OH)_2_D_3_ or dietary vitamin D supplementation were accompanied by decreased NF‐κB (p65) in KO intestinal epithelia.^(^ ^67^ ^)^ Suppression by 1,25(OH)_2_D_3_ of inflammatory cytokines was also observed using *RAB11A* KO Caco‐2 cells, suggesting that the observed effects of 1,25(OH)_2_D_3_ were likely mediated by epithelial cell intrinsic mechanisms.^(^ ^67^ ^)^ Further studies are needed to determine the pathways modulated by vitamin D/1,25(OH)_2_D_3_ in IECs and specific cell types that may be involved in the inflammatory bowel phenotype.

## Conclusion

The major target of vitamin D is the intestine. Intestinal effects of 1,25(OH)_2_D_3_ on intestinal calcium absorption are more complex than suggested by previous traditional models. Studies in *Trpv6* and *S100g* null mice indicate that our understanding of the vitamin D mediated intestinal calcium transport process remains incomplete. Recent studies suggest that vitamin D affects not a single entity, but rather a complex network of calcium‐regulating components is involved in 1,25(OH)_2_D_3_‐mediated active intestinal calcium absorption (e.g., calmodulin binding to TRPV6 and fine‐tuning TRPV6 activity, calcium binding to intracellular organelles as well as calbindin, and 1,25(OH)_2_D_3_ regulation of intestinal transporters of other ions that may also contribute to calcium transport activity). In addition to maintaining calcium homeostasis, vitamin D may also play other roles as a regulator of intestinal physiology. Compelling data from the laboratory, including studies in mouse models, provide evidence for 1,25(OH)_2_D_3_ effects independent of calcium regulation, which include the regulation of intercellular adhesion molecules to enhance barrier function, the regulation of intestinal stem cell function, cellular homeostasis of other divalent ions, the regulation of drug‐metabolizing enzymes, and anti‐inflammatory effects. Future studies are needed to identify the physiological significance of the control by vitamin D of novel intestinal vitamin D targets. Although further definition is also needed related to the contribution of the effects observed in the setting of human disease, there is convincing evidence in the laboratory of beneficial effects of 1,25(OH)_2_D_3_ in the intestine beyond intestinal calcium absorption, which include actions involved in xenobiotic metabolism and protection against intestinal inflammation.

## Conflict of Interest Disclosure

The author declares no conflict of interest.

### Peer Review

The peer review history for this article is available at https://publons.com/publon/10.1002/jbm4.10554.
